# Therapeutic Efficacy of Selenium Pre-treatment in Mitigating Cadmium-Induced Cardiotoxicity in Zebrafish (*Danio rerio*)

**DOI:** 10.1007/s12012-024-09910-0

**Published:** 2024-08-30

**Authors:** Rachael M. Heuer, Priscila Falagan-Lotsch, Jessica Okutsu, Madison Deperalto, Rebekka R. Koop, Olaedo G. Umeh, Gabriella A. Guevara, Md Imran Noor, Myles A. Covington, Delia S. Shelton

**Affiliations:** 1 Department of Marine Biology and Ecology, Rosenstiel School of Marine, Atmospheric, and Earth Science, University of Miami, Miami, FL 33149 USA; 2 Department of Biological Sciences, Auburn University, Rouse Life Sciences Building, Auburn, AL 36849 USA; 3 Department of Biology, University of Miami, 1301 Memorial Dr., Coral Gables, FL 33134 USA

**Keywords:** Cadmium, Selenium, Heart rate, Epicardial edemas, Zebrafish

## Abstract

Cardiovascular diseases are a rampant public health threat. Environmental contaminants, such as Cadmium (Cd), a toxic metal, are risk factors for cardiovascular diseases. Given that human exposure to Cd is increasing, there is a need for therapies to ameliorate Cd toxicity. Selenium (Se), an essential trace element, has been proposed to rescue the effects of Cd toxicity, with mixed effects. Se's narrow therapeutic window necessitates precise dosing to avoid toxicity. Here, we assessed the effects of various waterborne Cd and Se concentrations and sequences on cardiac function using zebrafish (*Danio rerio*). We showed that Cd induced pericardial edemas and modified heart rates in zebrafish larvae in a concentration-dependent manner. To identify the therapeutic range of Se for Cd-induced cardiotoxicity, zebrafish embryos were treated with 0, 10, 50, 100, 150, or 200 μg/L Se for 1–4 days prior to exposure to 2.5 and 5 μg/L Cd. We found that a 50 µg/L Se pre-treatment before 2.5 μg/L Cd, but not 5 μg/L Cd, reduced the prevalence of pericardial edemas and ameliorated Cd-induced bradycardia in zebrafish. Zebrafish exposed to 10 and 50 μg/L of Se for up to 4 days showed typical heart morphology, whereas other Se-exposed and control fish presented pericardial edemas. Longer Se pre-treatment durations led to fewer incidences of pericardial edemas. Overall, this study highlights the importance of optimizing Se concentrations and pre-treatment periods to harness its protective effects against Cd-induced cardiotoxicity. These findings provide insights into potential therapeutic strategies for reducing Cd-related cardiovascular damage in humans.

**Graphical Abstract:**

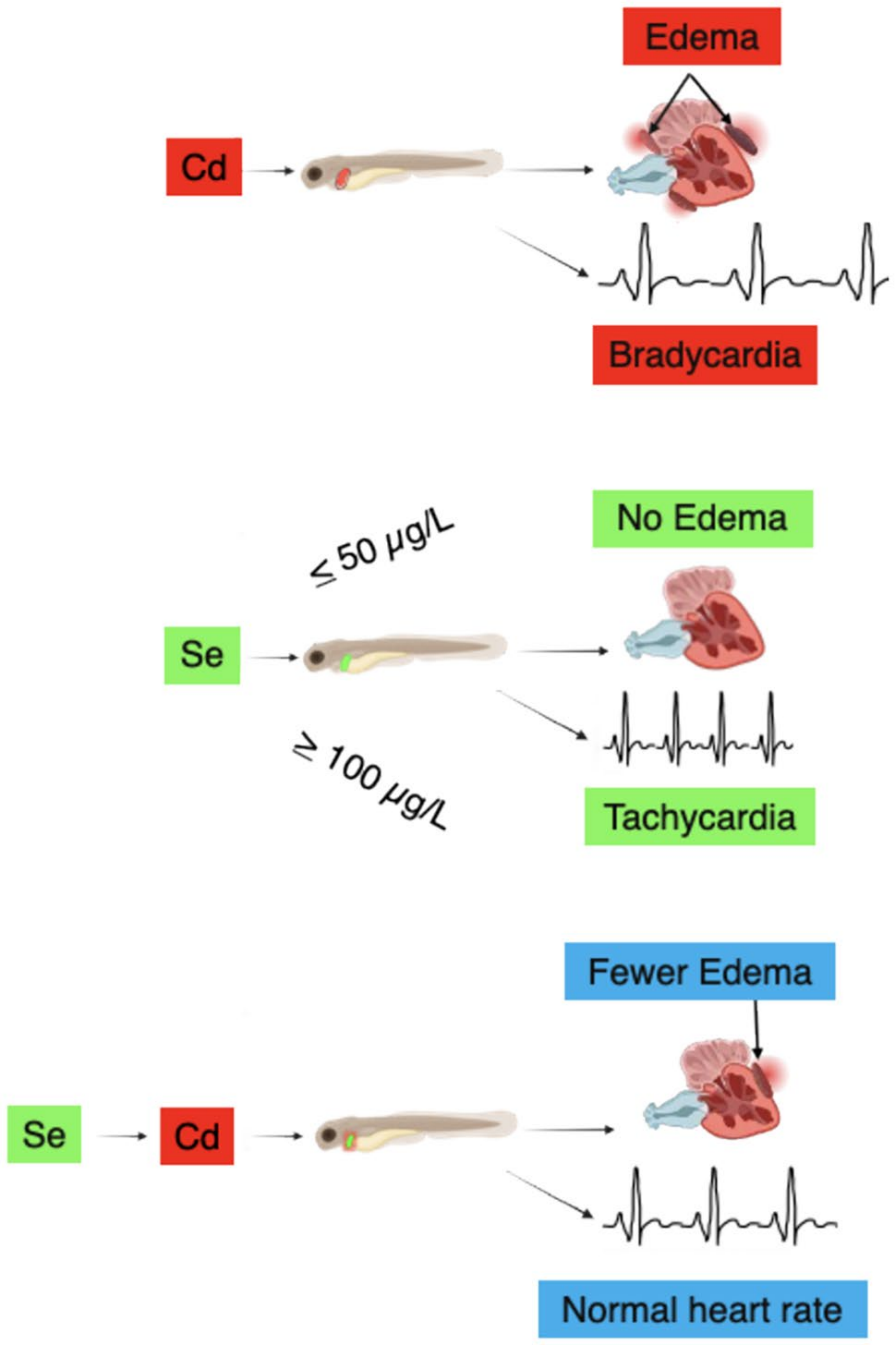

**Supplementary Information:**

The online version contains supplementary material available at 10.1007/s12012-024-09910-0.

## Introduction

Cardiovascular diseases (CVDs) have long been a critical public health issue and remain a leading cause of death worldwide [1]. According to the World Health Organization (WHO), CVDs account for an estimated 17.9 million deaths globally each year [2]. In recent years, exposure to environmental pollutants, such as fine particulate matter (PM2.5) in the air and toxic metals like cadmium, lead, and arsenic, has been strongly linked to the progression of CVDs and the incidence of cardiovascular events [3, 4]. In 2019, nearly 62% of all deaths related to environmental pollution were attributed to CVDs, according to the Global Burden of Disease research [5]. While air pollution is already recognized as a risk factor for CVDs, medical societies have not yet uniformly addressed vascular toxicity from contaminant metals despite epidemiological evidence linking chronic exposure to low and low-moderate levels of these metals [6].

Cadmium (Cd) is a trace metal present in the natural environment and is released into water and air during weathering processes [7–9]. However, anthropogenic activities such as mining release Cd into the environment at concentrations toxic to humans and ecosystems (Françoise [10]). Cd was recently ranked seventh on the Agency for Toxic Substances and Disease Registry substance priority list, underscoring the need to better understand its impacts on human health [11]. Routes of exposure include the extraction and processing of ore, processing of Cd-containing industrial waste, or through phosphate fertilizers (Françoise [10, 12, 13]). Non-occupational exposure can occur through cigarette smoking, or exposure through Cd-contaminated water or air [14]. However, the primary source of exposure tends to be from diet [15, 16]. Of particular concern is Cd’s long half-life, which can span decades [17].

The negative health impacts of Cd exposure in humans, including genotoxic damage, nephrotoxic injury, disruptions to calcium homeostasis, osteomalacia [18, 14, 19], and its classification as a known carcinogen (Hartwig, 2013), have been well-documented. Cd is known to target multiple organ systems and could predispose humans to increased risk of hypertension, Alzheimer’s disease, and impaired reproductive health [20, 9]. Increasing attention has focused on the impacts of Cd on the cardiovascular system. A recent population-based study reported a significant association between the blood levels of Cd and increased susceptibility to coronary heart disease [21], hypertension [22], and arterial disease [23]. While the association between Cd and cardiovascular disorders is becoming more established, the pathophysiological mechanisms require further examination [6].

Zebrafish serve as a well-established model for investigating how environmental pollutants impact development, morphology and the cardiovascular system in the lab [24–27]. Field studies of zebrafish and other fishes help us ground our metrics ethologically [28–31]. Cellular differentiation and cellular migration during development, and the electrical properties of zebrafish hearts, closely mirror patterns observed in mammals, making them valuable for studying physiological and molecular mechanisms underlying toxicant exposures [32, 27]. Their transparent early life stages allow for easy visualization of basic cardiovascular responses, including stroke volume, and heart malformations [33]. Various transgenic zebrafish lines enable visualization of cardiac malformations following toxicant exposures in live animals [32]. Furthermore, unlike mammals, zebrafish are capable of regenerating cardiac tissue following injury, and could provide valuable insight into how toxicants can impact these repair processes [34].

Cd is known to cause a wide array of impacts in larval and adult zebrafish [35, 25, 26, 36]. Various studies report lethal dose/concentration (LC/LD50) cadmium exposure in larval zebrafish: ~ 122–1730 μg/L [37], 168 μM [38], and these values tend to be water ion concentration, pH, and life stage-specific [37–39]. Similarly, sub-lethal impairments to the cardiovascular system vary across studies and are both concentration- and life-stage specific. Common themes are that Cd often leads to cardiac edema [38, 40, 41] and/or reduced heart rate (bradycardia) [42]. For example, in developing zebrafish, waterborne exposure to 1.0 μM Cd led to a reduced heart rate at 72 Hours Post-Fertilization (hpf), but not at 48 hpf [42]. Another study, testing a wider concentration response through 4 Days Post-Fertilization (dpf, 0.01–10 μM, 1.124–1124 μg/L waterborne Cd), showed tachycardia (increased heart rate) at 5 dpf [36]. However, when these same groups were later examined as adults after being reared in control water (8–10 months), zebrafish exposed to Cd during development exhibited an inconsistent concentration response, where bradycardia was observed at 1.0 μM Cd but not at lower and higher concentrations [36]. Here, we ask if the inconsistencies in bradycardia are due to morphological differences or other compensatory mechanisms.

Over the past decades, selenium (Se) has emerged as a potential therapeutic agent to counteract Cd-induced toxicity in various animal organs and tissues, including the heart [43–47]. Se is an essential trace element for many eukaryotes, including mammals and fish, primarily found in natural food sources as selenomethionine, selenocysteine, selenium-methylselenocysteine (organic forms), and selenate (inorganic form) [48–50]. In its form as selenocysteine, Se is incorporated into proteins to form selenoproteins, which are crucial for maintaining redox balance in cells [51, 52]. At low levels, Se exhibits potent antioxidant activity by upregulating selenoproteins such as glutathione peroxidase (GPx) and thioredoxin reductase (TrxR). These enzymes help eliminate reactive oxygen species (ROS) and suppress oxidative stress-mediated cell damage, a major mechanism underlying Cd-induced toxicity and related to the etiology of several chronic diseases, including CVD [53–56]. However, other mechanisms have been proposed to explain the role of Se in alleviating Cd-induced cell damage. Se can sequester Cd, forming a biologically inert compound, thereby reducing Cd accumulation in cells and tissues. This sequestration has been suggested as the major mechanism of action of Se against Cd toxicity [57]. The activation of the Nrf2 pathway, a master regulator of cellular redox homeostasis, has also been proposed as a mechanism by which Se counteracts Cd-induced oxidative stress, thereby mitigating its toxicity [58]. However, Se has one of the narrowest therapeutic windows, with a fine line between its protective and toxic effects. Lethal concentrations of selenium during early life stage exposures in zebrafish range from 1 μM to 1 mM, and are dependent on whether selenium is in an organic or inorganic form [59]. Although experimental evidence has demonstrated the protective role of Se against heart damage, including the cardiotoxicity induced by Cd [60–62], other observational studies and randomized trials have linked Se to an increased risk of cardiovascular disease even at low concentrations [63–66]. These inconsistent findings highlight the need for additional studies to gain new insights into the role of Se in cardiac health and its beneficial doses.

The overall goal of this study was to determine whether or not prophylactic exposure to Se served to alter Cd-induced alterations to cardiac phenotypes. This was achieved by assessing the presence of pericardial edema and measuring heart rate across various combinations of Cd and Se concentrations in developing zebrafish. These endpoints were measured in controls, Cd-exposed, Se-exposed, and Se to Cd-transferred zebrafish larvae (5 dpf). In addition, we sought to determine if Se protective effects were dependent on the length of Se pre-exposure. We hypothesized that Cd exposure would lead to concentration-dependent pericardial edema and bradycardia, as has been noted in other studies on zebrafish [38, 42, 40, 41]. Further, we hypothesized that Se would have cardioprotective effects, but these effects were likely to be dependent on Se concentration and how long zebrafish were pre-exposed.

## Methods

### Subjects

We used a wild-type, outbred, 5D strain of zebrafish. The adult fish were housed under standard laboratory conditions: pH range of 7.2–8.2, conductivity range of 513.8–708.2 µS, nitrate range of 0.0–0.5 ppm, temperature range of 23.6–29.0 °C, ammonia range of 0.0–0.4 ppm and a light–dark cycle of 14:10 h. To generate embryos, we placed female and male adult zebrafish in a spawning tank that separated the sexes with a divider. The divider was then removed the next morning to ensure that embryos were similar in age. All collected embryos were bleached with 0.0003% sodium hypochlorite dissolved in reverse osmosis water and then placed in a petri dish containing embryo media (E3) with a maximum of 50 embryos per petri dish and placed in an incubator regulated to maintain a temperature of 28.5 ± 1 °C. All water that housed the fish including laboratory and E3 media had Cd that was below the detection limit of < 0.1 µg/L (US EPA Method 200.8). All experiments were done at the University of Miami under protocols #21-194LF and #22-028LF approved by the Institutional Animal Care and Use Committee (IACUC).

### Pericardial Edema Experimental Design

**Fig. 1:**
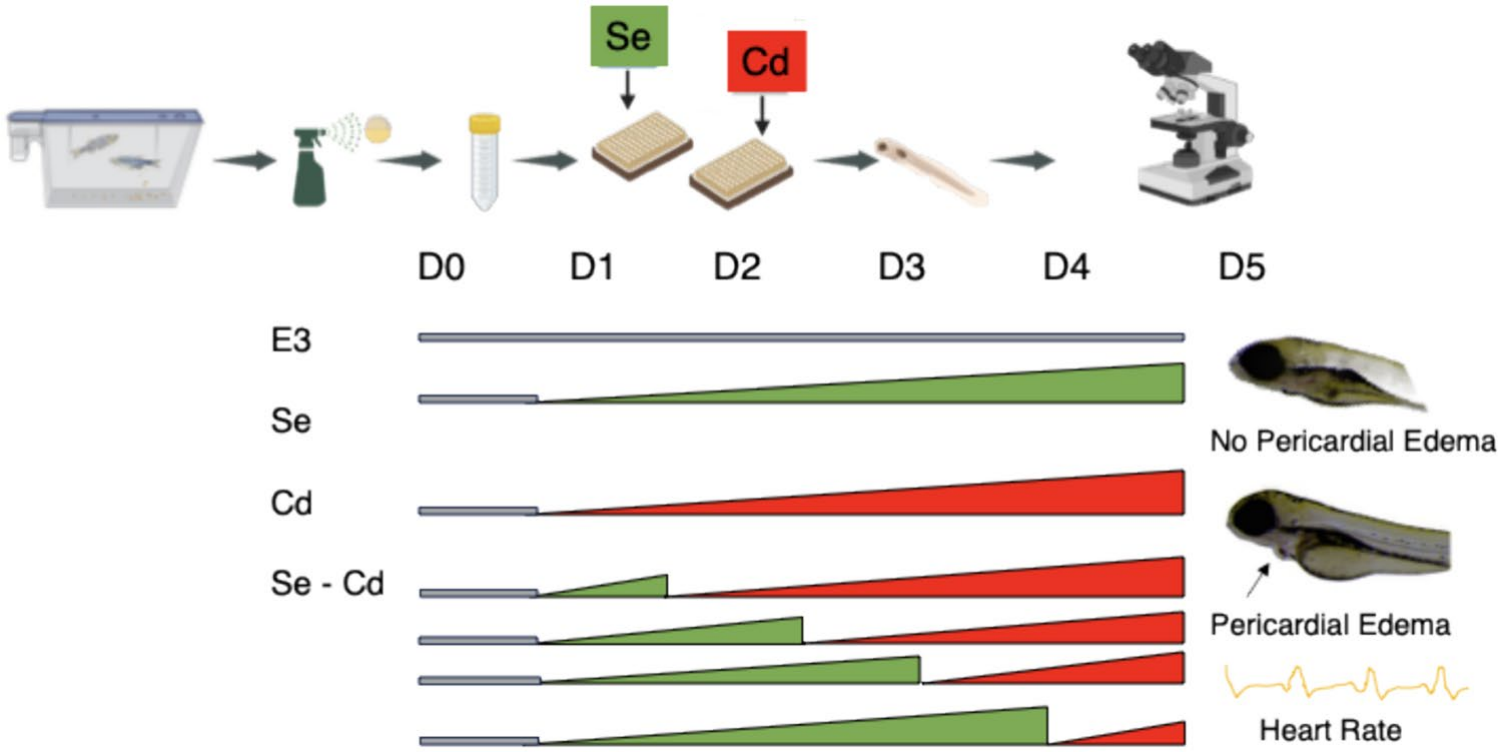
The experimental design included spawning adult zebrafish, collecting the embryos and bleaching them and placing embryos into well-plates by 6 hpf. We placed a subset of the embryos into E3 media (control) and others were placed in Se and Cd dissolved in E3 media. On days 1–4, zebrafish were then transferred to E3, Se, or Cd. On day 5, we assessed the presence (or absence) of pericardial edemas and measured heart rate

At ≤ 6 hpf, zebrafish embryos were transferred into well plates containing E3 media (control), while others were placed in Selenious acid (Cas # 7783–00-8, Thermo Scientific) or Cadmium Chloride (Cas # 10,108-64-2, Sigma Aldrich) dissolved in E3 media. To assess the therapeutic potential of Se for Cd toxicity, we exposed zebrafish from Day 0 (D0) to Day 5 (D5) to 0, 10, 50, 100, 150, 200 µg/L of Se and/or 0, 2.5 or 5 µg/L of Cd. These Se and Cd concentrations reflect human relevant exposures [67]; see discussion for more details). Zebrafish embryos exposed solely to E3 media for 5 days (~ 120 hpf) served as our control (Fig. 1). From days 1 to 4, zebrafish were transferred from Se to Cd solutions each day. Zebrafish controls that were exposed to E3 media, Se only, and Cd only were transferred each day to well plates containing the same treatment solutions to account for handling. Controls and test treatments were added to 96-well plates with one embryo per 300 µg/L of test solution. Up to 8 replicates for each treatment and control were added to well plates in block randomized order to be assessed at the same time. The embryos transferred from Se to Cd were rinsed three times in Cd solution prior to transfer. On day 5, we assessed the presence (1) or absence (0) of pericardial edemas in zebrafish larvae by viewing each fish under a Zeiss Discovery v20 microscope (Fig. 1).

### Cardiotoxicity Experimental Design

**Fig. 2:**
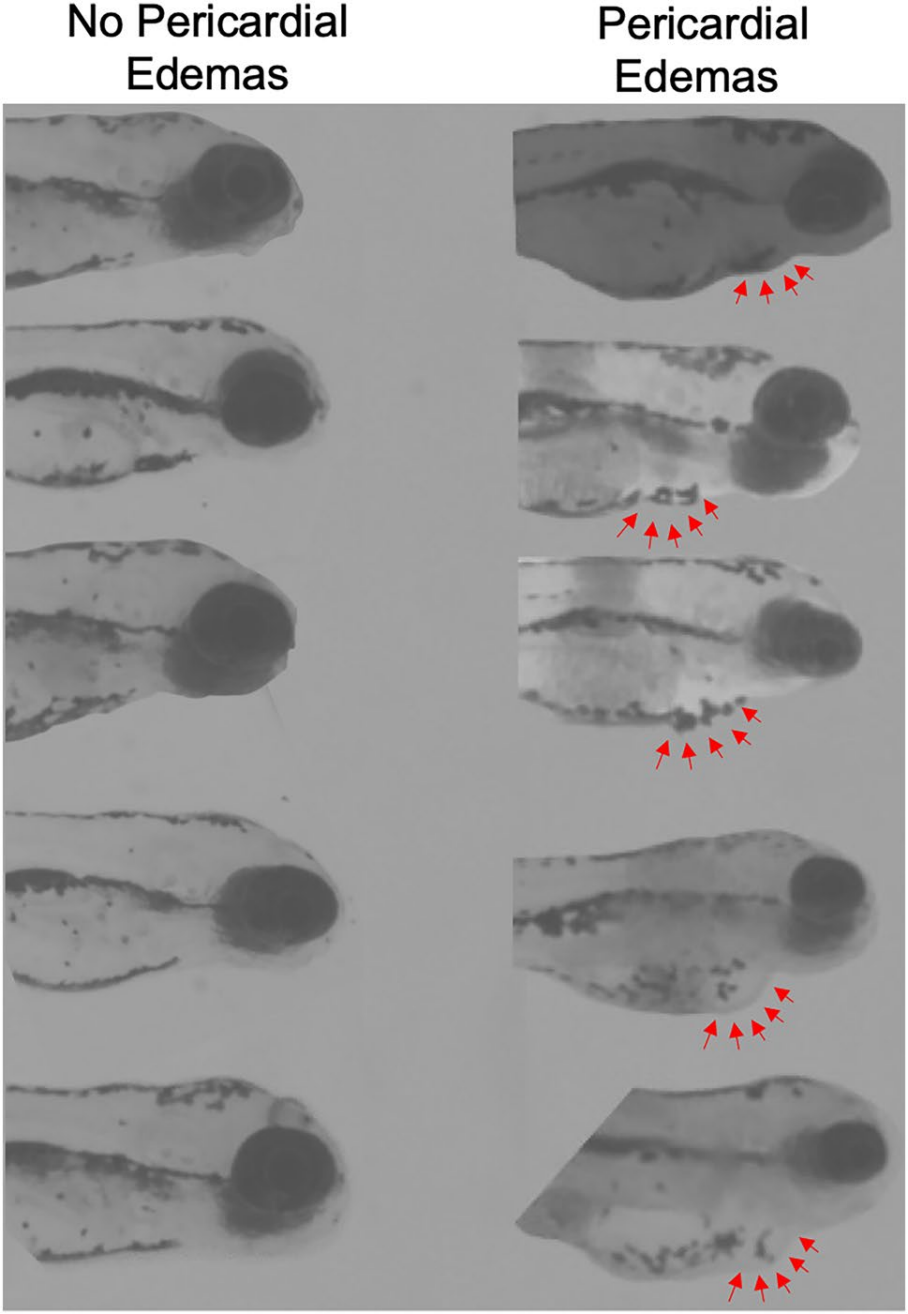
Representative images of fish exhibiting no pericardial edemas and fish exhibiting pericardial edemas. The pericardial edemas are indicated with red arrows. The fish are viewed at ~ 30 × magnification

To assess the effects of Se pre-exposure on the Cd-induced toxicity on heart rate, we exposed embryonic zebrafish to two different concentrations of Cd (2.5 µg/L or 5 µg/L), and 100 µg/L of Se in different combinations (Fig. 1). The exposure paradigm is similar to the pericardial edema experimental design with these noted differences. On day 1, zebrafish embryos exposed to 100 µg/L of Se were transferred to 2.5 µg/L or 5 µg/L of Cd. Zebrafish embryos exposed to 100 µg/L of Se were transferred to 5 µg/L of Cd on day 4. On day 5, we measured the heart rate of the zebrafish larvae by counting the number of heart beats that occurred in 10 s while the fish were immobilized on a slide using methylcellulose under a Nikon light microscope (Model 167,511). For a subset of fish, we noted the presence or absence of pericardial edemas (Fig. 1, 2).

### Analysis

We used Kruskal–Wallis chi-squared tests to compare fish exposed to Se and Cd across different concentrations and days of transfer to identify differences in the presence or absence of pericardial edemas. We fit the chi-squared models using the “kruskal.test” function in the stats package. For Kruskal–Wallis tests that reached statistical significance, we followed up with Dunn’s Multiple Comparisons post-hoc tests using “dunnTest” function in the chisq.posthoc.test package. To identify differences in heart rate across treatment groups, we used a one-way analysis of variance (ANOVA). We fit the ANOVA models using the “aov” function in the base package followed by the “Anova” with type III sums of squares function in the car package for unbalanced ANOVAs [68]. We examined the residuals to determine if square-root or log transformations were necessary. For ANOVAs that reached statistical significance, we followed with Tukey HSD tests. For comparisons involving heart rate between pairs of groups, we used independent samples t-test (two-tailed). We applied Welch’s correction when data were not equal in variance. Our alpha level was 0.05. We used R for all statistical tests [69]. Graphs were made using the “base” package in R version 4.2.3 [69].

## Results

### Lower Concentrations, But Not Higher Concentrations of Se Lead to Fewer Incidences of Pericardial Edemas in Zebrafish Larvae

**Fig. 3:**
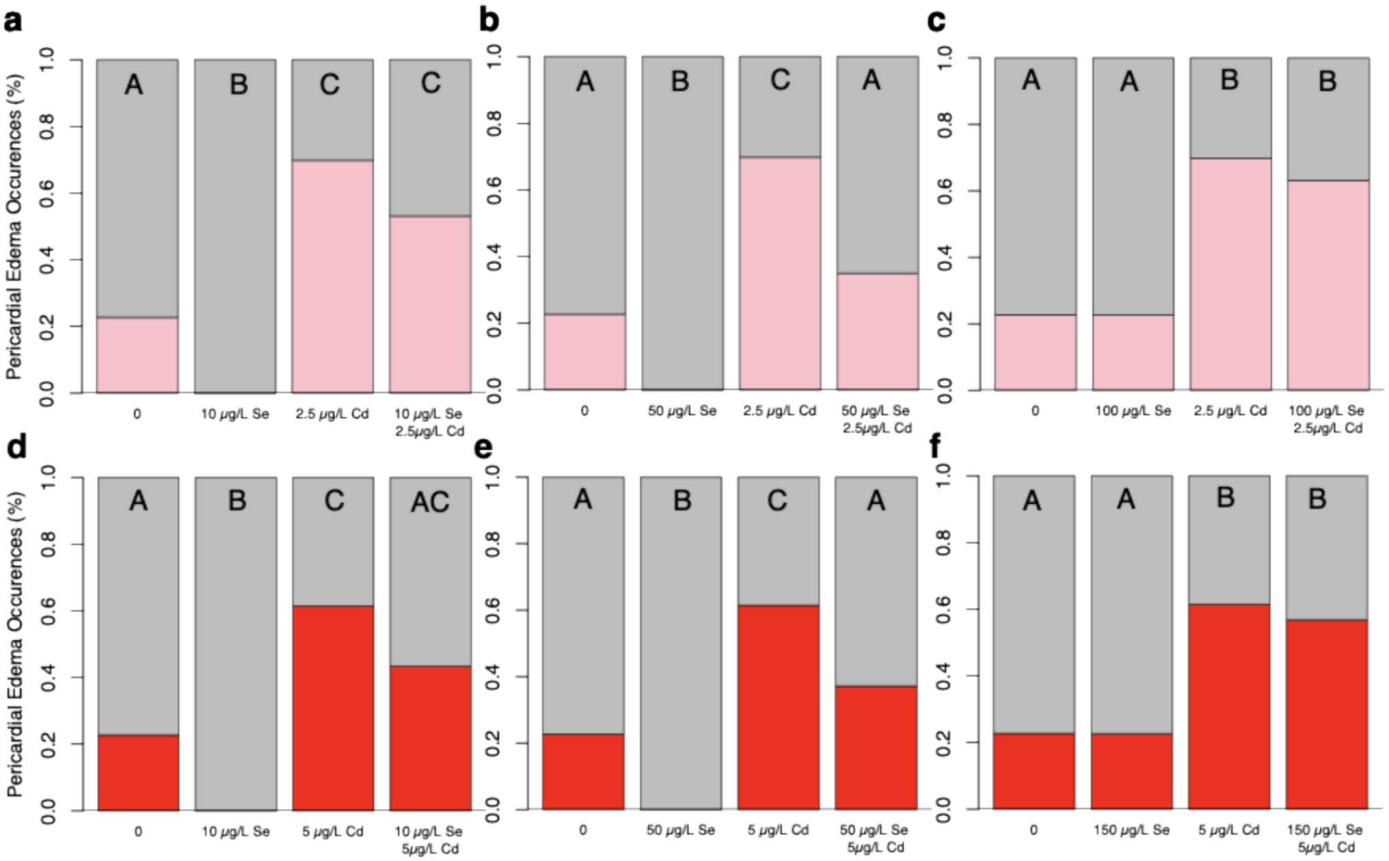
Effects of Se Pre-exposure on Cd-induced pericardial edemas in zebrafish larvae (5 dpf). Incidence of pericardial edemas in zebrafish exposed to E3 media (control; *n* = 167), 2.5 µg/L of Cd (*n* = 169) compared to those exposed to **a** 10 µg/L of Se (*n* = 31), fish pre-treated with 10 µg/L of Se prior to 2.5 µg/L of Cd (*n* = 32); **b** 50 µg/L of Se (*n* = 40), and fish pre-treated with 50 µg/L of Se prior to 2.5 µg/L of Cd (*n* = 40); **c** fish exposed to 100 µg/L of Se prior to Cd (n = 155) and 100 µg/L of Se (*n* = 159). Incidences of pericardial edemas in fish exposed to E3 media (control; *n* = 167) and 5 µg/L of Cd (*n* = 166) compared to those treated with **d** 10 µg/L of selenium (*n* = 31) and fish pre-treated with 10 µg/L of Se prior to Cd (*n* = 30); **e** fish treated with 50 µg/L of selenium (*n* = 40) and fish pre-treated with 50 µg/L of Se prior to Cd (*n* = 38); **f** Fish exposed to 100 µg/L of Se prior to Cd (*n* = 156) and those exposed to 100 µg/L of Se (*n* = 159). The letters above each bar show the results of Dunn’s tests. Bars that have the same letter do not significantly differ from each other (*P* > 0.05)

Zebrafish exposed to 2.5 µg/L of Cd exhibited approximately three times more pericardial edemas than unexposed Cd fish (Fig. 2a; *H*(3, *n* = 399) = 101.78, *p* < 0.0001). Among fish exposed to 2.5 µg/L of Cd, those pre-treated with 10 µg/L of Se showed a trend towards 16.7% fewer pericardial edemas, although this difference did not reach statistical significance (Tukey HSD, *p* = 0.08). Zebrafish larvae treated with 10 µg/L of Se displayed fewer pericardial edemas compared to control fish. Zebrafish pre-treated with 50 µg/L of Se before exposure to 2.5 µg/L of Cd showed less than half the percentage of pericardial edemas compared to those exposed to Cd alone and exhibited a percentage of pericardial edemas similar to controls (Fig. 3b). Notably, zebrafish placed in E3 media (controls) for 5 days showed a 22.8% incidence of pericardial edemas, whereas fish exposed to 50 µg/L of Se showed no evidence of pericardial edemas (Fig. 3b). This difference led to a significant difference in pericardial edemas across the four treatment categories for fish treated with 50 µg/L Se and 2.5 µg/L of Cd (*H*(3, *n* = 416) = 109.26, *p* < 0.0001). Similar results were found for fish pre-treated with 50 µg/L of Se prior to a 5 µg/L Cd exposure (Fig. 3e; *H*(3, *n* = 411) = 79.79, *p* < 0.0001). Control fish show 22.7% more incidences of pericardial edemas than fish treated with 50 µg/L of selenium (Tukey HSD, *p* < 0.05). Fish exposed to ≥ 100 µg/L of Se showed a similar frequency of pericardial edemas as those exposed to 2.5 µg/L or 5 µg/L of Cd (Fig. 2c, f, Supplementary figures). Controls and zebrafish exposed to ≥ 100 µg/L of Se showed comparable incidences of pericardial edemas (Fig. 3f). Fish exposed to 2.5 µg/L or 5 µg/L of Cd with or without a ≥ 100 µg/L Se pre-treatment, displayed nearly three times as many incidences of pericardial edemas compared to fish exposed to ≥ 100 µg/L Se and controls. This difference led to a significant difference in pericardial edemas across the four treatment categories for fish treated with 100 µg/L Se and 2.5 µg/L of Cd (*H*(3, *n* = 650) = 128.35, *p* < 0.0001) or 5 µg/L Cd (*H*(3, *n* = 648) = 86.85, *p* < 0.0001).

### Selenium Has a Narrow Therapeutic Range

**Fig. 4:**
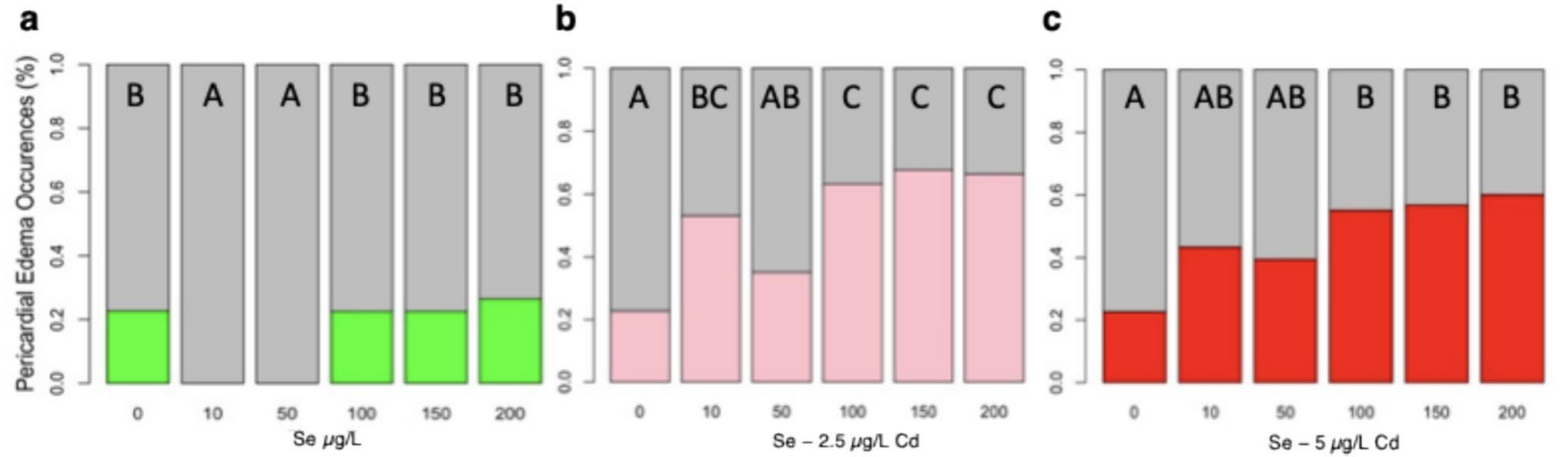
Effects of Se pre-exposure on Cd-induced pericardial edemas in zebrafish larvae (5 dpf). **a** The percentage of zebrafish larvae expressing pericardial after exposure to 0 (n = 167), 10 (*n* = 31), 50 (*n* = 40), 100 (*n* = 159), 150 (*n* = 128) and 200 (*n* = 128) µg/L of Se. **b** The percentage of zebrafish larvae expressing pericardial after pre-treatment with 0 (n = 167), 10 (*n* = 32), 50 (*n* = 40), 100 (*n* = 155), 150 (*n* = 130) and 200 (*n* = 128) µg/L of Se prior to 2.5 µg/L of Cd. **c** The percentage of zebrafish larvae expressing pericardial after pre-treatment with 0 (n = 167), 10 (*n* = 30), 50 (*n* = 38), 100 (*n* = 156), 150 (*n* = 132) and 200 (*n* = 128) µg/L of Se prior to 5 µg/L of Cd. The letters above each bar show the results of Dunn’s tests. Bars that have the same letter do not significantly differ from each other (*P* > 0.05)

Zebrafish larvae (5 dpf) exposed to 10 and 50 µg/L of Se for 5 days display no pericardial edemas, whereas 22.7–26.6% of Control zebrafish and fish exposed to ≥ 100 µg/L of Se show pericardial edemas (Fig. 3a). These differences in the Se-exposed groups led to a significant difference across treatments *H*(5, *n* = 653) = 22.02, *p* = 0.0005. Among zebrafish larvae pre-treated with Se prior to a 2.5 µg/L Cd exposure, 35% of those treated with 50 µg/L of Se showed pericardial edemas, which is 12% more than control fish and 18% fewer than those pre-treated with 10 µg/L of Se (Fig. 4b). Zebrafish pre-treated with higher concentrations of Se, ≥ 100 µg/L of Se, showed 1.8 times more incidences of pericardial edemas than those pre-treated with 50 µg/L of Se. Difference across treatments were significantly different, *H*(5, *n* = 652) = 93.04, *p* < 0.0001. Zebrafish pre-treated with Se prior to a 5 µg/L Cd exposure showed more pericardial edemas than control fish (Fig. 4c). Specifically, 43% and 39% of zebrafish larvae pre-treated with 10 µg/L and 50 µg/L of Se prior to Cd exposure showed pericardial edemas, respectively, whereas those treated with higher concentrations showed 12–20% more pericardial edemas. This difference led to a significant difference across treatments for 5 µg/L Cd-exposed pre-treated with Se, *H*(5, *n* = 651) = 58.51, *p* < 0.0001. Dunn’s post-hoc test revealed that all fish pre-treated with Se prior to a 5 µg/L of Cd had statistically similar occurrences of pericardial edemas (*p* > 0.05).

### Longer Se Pre-treatments Reduce Incidences of Pericardial Edemas in Cd-Exposed Zebrafish Larvae

**Fig. 5:**
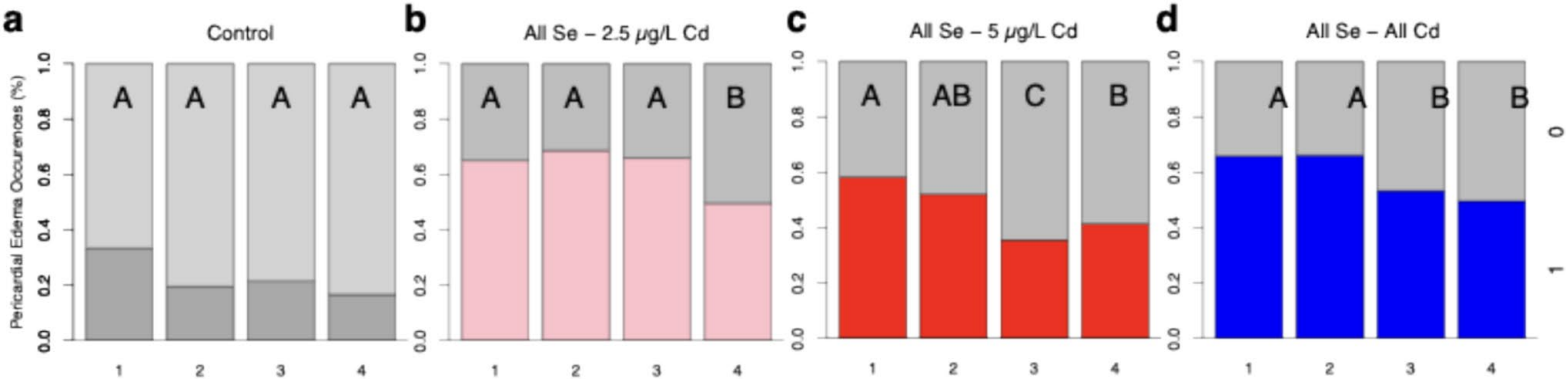
Impact of Se exposure duration on Cd-Induced pericardial edemas in zebrafish larvae. **a** Occurrences of pericardial edemas in control fish transferred to E3 media on days 1—4 (n = 41–42). **b** Occurrences of pericardial edemas in zebrafish larvae pre-exposed to Se for 1–4 days prior to transfer to 2.5 µg/L Cd (*n* = 120–123). **c** Occurrences of pericardial edemas in zebrafish pre-exposed to 100 µg/L of Se 1–4 days prior to being transferred to 5 µg/L of Cd (*n* = 156–167). **d** Composite figure showing fish pre-exposed to 100 µg/L of Se for 1–4 days and then transferred to 2.5 µg/L or 5 µg/L Cd (*n* = 236–246). The letters above each bar show the results of Dunn’s tests. Bars that have the same letter do not significantly differ from each other (*P* > 0.05)

Control fish transferred from well plates on days 1–4 show similar percentages of pericardial edemas. We found that 33% of control fish transferred on 1 dpf show pericardial edemas and 17–21% of control fish transferred on subsequent days experience pericardial edemas (Fig. 5a). These observations did not lead to a significant difference in pericardial edemas across days transferred (*H*(3, *n* = 167) = 3.82, *p* = 0.28). Zebrafish larvae pre-treated with Se for 1–3 days prior to a 2.5 µg/L Cd express at least 15% more pericardial edemas than those pre-treated for 4 days with Se (Fig. 5b). These differences led to a significant difference in pericardial edemas across treatments *H*(3, *n* = 485) = 11.37, *p* = 0.01. Zebrafish pre-treated with Se for 1 and 2 days prior to a 5 µg/L Cd exposure exhibit 58.2% and 51.9% incidence of pericardial edemas, respectively (Fig. 5b). Fish pre-treated with Se for 3 and 4 days show fewer pericardial edemas, with those pre-treated for 3 days showing 22.6% fewer pericardial edemas compared to the 1-day pre-treatment group. These observations led to a significant difference in pericardial edemas across treatments, *H*(3, *n* = 651) = 20.46, *p* < 0.0001. Overall, a Se pre-treatment duration of 3–4 days reduced the occurrence of pericardial edemas in zebrafish larvae by 16.5% compared to those pre-treated for 1–2 days (*H*(3, *n* = 969) = 21.78, *p* < 0.0001).

### Selenium Pre-treatment Ameliorates Cd-Induced Bradycardia in 2.5, But Not in 5 µg/L Cd-Exposed Zebrafish

**Fig. 6:**
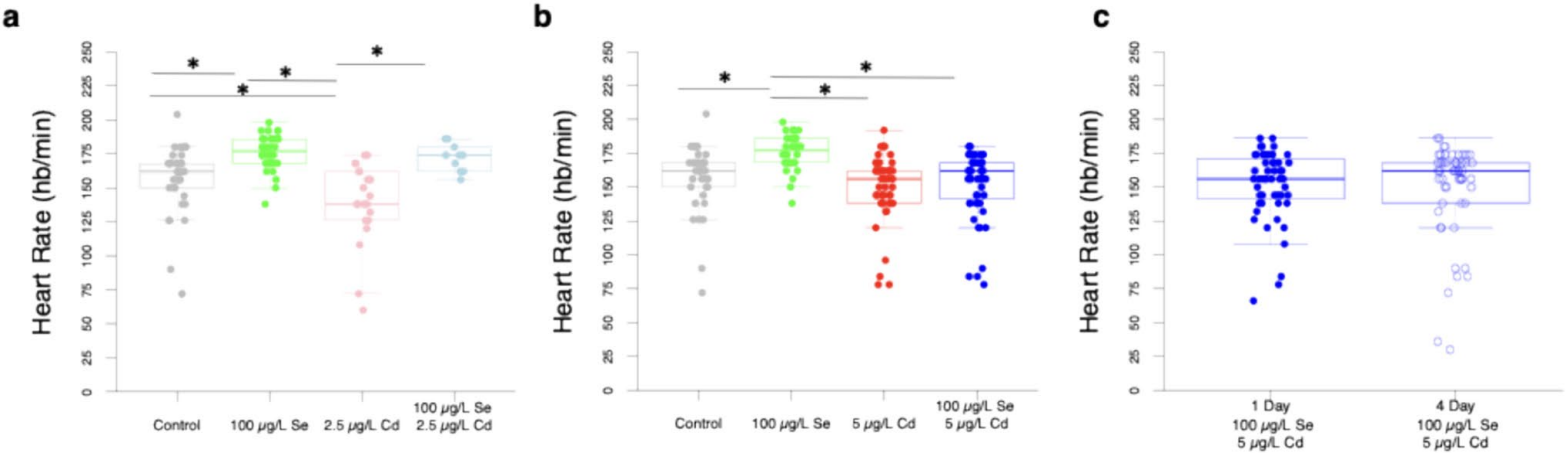
Se and Cd have opposite effects on zebrafish larvae heart rate. **a** Fish exposed to 100 µg/L of Se show elevated heart rates, whereas those exposed to 2.5 µg/L of Cd exhibit lower heart rates compared to control fish (*n* = 10–37). Fish pre-treated with 100 µg/L of Se prior to 2.5 µg/L of Cd exposure and control fish show similar heart rates. **b** Fish exposed to 5 µg/L of Cd with and without a 100 µg/L of Se pretreatment have heart rates similar to control fish (*n* = 34–56). **c** Fish exposed to 100 µg/L of Se for 1 and 4 days prior to 5 µg/L of Cd exposure display similar heart rates (*n* = 66, *n* = 48, respectively). Box-and whisker plots overlaid with individual fish represented as circles. *P < 0.05, Tukey HSD

Se induces tachycardia and Cd’s effect on heart rate is concentration dependent. Zebrafish larvae treated with 100 µg/L of Se and 2.5 µg/L of Cd display bradycardia (Fig. 6a). Fish exposed to 100 µg/L of Se showed 10.7% increase in heart rate compared to control fish. In contrast, fish exposed to 2.5 µg/L of Cd exhibited an 11.8% decrease in heart rate compared to control fish. The 21.3% difference between Se-exposed and Cd-exposed zebrafish was mitigated when fish were pre-treated with 100 µg/L of Se 1 day prior to Cd exposure. Fish exposed to 2.5 µg/L of Cd after 100 µg/L of Se pre-treatment show heart rates similar to control fish at 5 dpf. This difference in heart rates across treatments led to a significant difference (*F*(3, 98) = [15.25], *p* < 0.0001). Fish exposed to 5 µg/L of Cd, with or without a pre-treatment with 100 µg/L of Se (M = 24.8, SE = 0.61), display heart rates similar to control fish (M = 26.19, SE = 0.69). Fish exposed to 100 µg/L of Se experience 15.4% and 13.4% faster heart rates than those exposed to 5 µg/L of Cd and fish pre-treated with 100 µg/L of Se for 1 day prior to exposure to 5 µg/L of Cd, respectively. This observation led to a significant difference in heart rate across treatments (*F*(3, 170) = [10.32], p < 0.0001). Fish pre-treated with 100 µg/L of Se for 1 day and 4 days prior to 5 µg/L of Cd exposure have similar heart rates (Fig. 6c, t(79.35) = 0.94, *p* = 0.35).

### 5 µg/L, But Not 2.5 µg/L Cd-Exposed Zebrafish Larvae with Pericardial Edemas Have Lower Heart Rates Than Those Without Pericardial Edemas

**Fig. 7:**
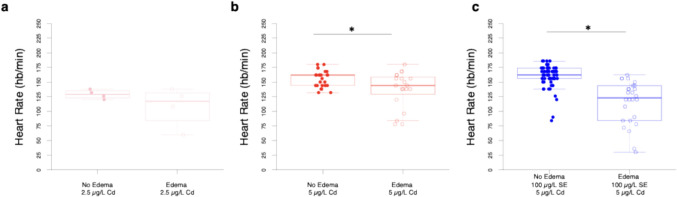
For higher concentrations of Cd, zebrafish larvae with pericardial edemas have lower heart rates. **a** Heart rate of fish exposed to 2.5 µg/L of Cd with and without the presence of pericardial edemas (*n* = 4 per condition). **b** Heart rate of dish exposed to 5 µg/L of Cd with (*n* = 20) and without (*n* = 25) pericardial edemas. **c** Fish exposed to 100 µg/L Se prior to 5 µg/L of Cd with (*n* = 26) and without (*n* = 62) pericardial edemas. Individual fish are represented as circles with closed circles representing fish without edemas and open circles representing fish with edemas. Box-and whisker plots overlaid with individual fish represented as circles. *P < 0.05, t-Test

The influence of pericardial edemas on the heart rate of zebrafish larvae is dependent on the Cd concentration. Fish exposed to 2.5 µg/L of Cd exhibit a 16.3% difference in heart rate depending on the presence of pericardial edemas, but this difference did not reach statistical significance (Fig. 7a, t(3.31) = 1.19, *p* = 0.31). Fish exposed to 5 µg/L of Cd that have edemas show 11.6% fewer beats per min (bpm) than fish without edemas (Fig. 7b, t(25.52) = 2.42, *p* = 0.02). Fish pre-treated with 100 µg/L of Se prior to 5 µg/L of Cd exposure with edemas display 27.8% fewer bpm than those without edemas (Fig. 7c, t(31.01) = 5.79, *p* < 0.001).

## Discussion

The primary objective of this study was to assess whether prophylactic Se exposure could modify Cd-induced changes in cardiac phenotypes observed in zebrafish larvae (5 dpf). This was achieved by evaluating pericardial edemas and heart rate across various concentration combinations of control, Cd-exposed, Se-exposed, and Se-Cd transferred animals. We also examined if the duration spent in the Se prophylactic treatment influenced Cd toxicity. Our findings confirm that Cd causes pericardial edemas in zebrafish and reduces their heart rate, and that Se can attenuate or rescue these phenotypes. However, the potential therapeutic effect of prophylactic Se exposure is both concentration- and time-dependent, with maximal protective effects below 50 μg/L of Se and with longer pre-exposure periods during the zebrafish developmental period (3–4 days).

As commonly observed in prior studies [38, 40, 41], exposure to Cd led to pericardial edemas at both 2.5 μg/L and 5.0 μg/L (Fig. 2), and bradycardia at 2.5, but not 5.0 μg/L (Fig. 5). Previous research has documented inconsistent responses in heart rate following developmental Cd exposure, with some showing bradycardia and tachycardia within the same study [36]. Proposed mechanisms underlying tachycardia include increased apoptosis triggering a compensatory baroreflex response or stress response [36]. Observations of bradycardia have been attributed to cardiac damage, pericardial edema, altered action potentials, or permanent alterations in sympathetic tone following Cd exposure [40, 36]. Notably, deviations from the control response could represent lower cardiac performance, which could ultimately impact fitness or increase energetic costs associated with compensatory mechanisms. We found that pericardial edemas tended to correlate with bradycardia at 5.0 μg/L Cd, but not at 2.5 μg/L (Fig. 7), possibly due to the small sample size for the 2.5 µg/L Cd group. More studies pairing heart rate measurements with other cardiovascular metrics can be insightful, as seen in [40], where developmental Cd exposure led to increased pericardial edema, reduced heart rate, increased stroke volume, and cardiac output in zebrafish at 96 hpf, while no changes were noted in ejection fraction. Authors in this study suggested that increased stroke volume acted as a compensatory mechanism in response to lower heart rate [40]. As suggested by [40], changes in cardiac function are a product of exposure regimes and could be due to a number of factors including changes in genes that encode Na^+^/K^+^ ATPase, myosin heavy chain, L-type Ca^2+^ channels. The effects may be dependent on temperature and the activation of the oxidative stress pathways. In addition to obtaining a better understanding of the mechanisms underlying Cd-induced cardiotoxicity, it is also important to explore therapeutic interventions. While some evidence suggests that interventions that reduce Cd burden may alleviate cardiovascular impacts, more evidence is needed [70].

Our study demonstrated the potential of Se as a therapeutic agent against the adverse effects of Cd on the heart of zebrafish in a concentration-dependent manner. Exposure to Se (100 μg/L for 1 day) rescued Cd-induced heart rate changes observed in zebrafish larvae (Fig. 6). Reduced heart rate in response to Cd was noted at 2.5 μg/L but not at 5.0 μg/L. While this result was unexpected initially, coupling the heart rate measure with pericardial edemas assessments revealed that fish with edemas showed significantly lower heart rates than those without edemas (Fig. 6). Further, it is important to note that heart rate is just one component of the cardiovascular system that can compensate for Cd-induced impacts. For example, observations of reduced heart rate were accompanied by increased stroke volume in developing zebrafish exposed to 16.7 μM (3 mg/L) Cd for 96 h, which ultimately led to an increase in cardiac output [40]. Additionally, prior exposure to Se (3–4 days) reduced the occurrence of pericardial edemas caused by Cd at both 2.5 and 5 μg/L, indicating that Se might be used as a preventive treatment for Cd toxicity when Se dosage and time are appropriate (Fig. 5). Moreover, given that the treatment with Se (up to 50 μg/L alleviated the elevated incidence (22.7%) of pericardial edema in control zebrafish, it is likely that our controls may have experienced a selenium-related deficiency. This observation does support the conclusion that certain Se concentrations could offer cardioprotective effects, and that potential deficiencies should be considered if such pathologies are observed in zebrafish colonies; confirmation that the control fish are selenium deficient requires more advanced analytical methods to assess elemental body burdens (e.g., Inductively Coupled Plasma-Mass Spectrometry). Previous studies have highlighted the cardioprotective effects of both organic and inorganic Se compounds, as well as Se nanoparticles (Nano-Se), in alleviating Cd-induced oxidative stress, programmed cell death, and inflammation [61, 71, 72]. In addition, selenium deficiency has been related to cardiovascular pathologies in epidemiological studies (see review, [73]. Thus, there is potential synergy in the pathobiology of humans and zebrafish in the presence (or absence) of selenium.

The same concentration of Se (100 μg/L) that mitigated Cd effects on heart rate also induced pericardial edemas and tachycardia (increased heart rate) in zebrafish larvae, indicating acute Se toxicity (Fig. 6). The effects of Se on heart health are complex and remain controversial. It has been suggested that Se may induce cardiotoxicity in a dose-dependent manner [65, 74, 75]. Nevertheless, there is no consensus about reference levels for Se intake [76] due to little evidence on dose–response relationships between Se and health outcomes. The normal range of Se is based on the Se levels in healthy populations worldwide and varies among different regions [77–79]. In the United States, the intake of Se is considered high compared to other countries with an average of 116 μg/daily in people aged 2 years and older (in France, Se intake is 64 ± 14 μg/day in adults) (NIH Office of Dietary Supplements, 2018). This average is higher than the concentration of Se related to cardiac toxicity effects in our study. A recent prospective cohort study in a representative sample of the United States population with non-alcoholic liver disease using The National Health and Nutrition Examination Survey (NHANES)-III (1988–1994) illustrated the non-linear, U-shaped dose–response relationship between dietary Se intake and cardiovascular mortality [80]. This result indicates that both low and high levels of Se are detrimental to cardiac function.

## Conclusions

Our study found that pre-exposure to Se can ameliorate negative cardiac impacts induced by Cd exposure in larval zebrafish, however, this attenuation was both dose and time-dependent. Our findings suggest optimal protective effects occur with longer Se pre-exposures and at concentrations below 50 μg/L. Our study suggests that more research focused on the investigation of the impact of Se and Cd at different concentrations on cardiac health is needed. In future studies, we will explore how Se at different concentrations exerts beneficial or toxic effects on the heart at multiple biological levels of organization (molecular, cellular, and physiological, intact animal) using both zebrafish and in vitro new approach methods such as primary cells and/or human-induced pluripotent stem cell-derived cardiomyocytes as models for cardiotoxicity testing [81, 82]. Identifying key gaps in our knowledge about the positive and negative effects of Se on heart health and its therapeutic potential to alleviate Cd-induced cardiotoxicity could help mitigate cardiovascular morbidity. In future investigations, it would be useful to overlay the ontogeny of cardiovascular system development with physiological measures to better understand both mechanisms of Cd cardiotoxicity and the therapeutic potential of Se pre-exposure [27]. Concurrent examination of physiological endpoints throughout development paired with the mRNA expression of cardiac-development related genes such as tbx5, gata4, nxk2.5, myh6, my17, andtnnt2, and chd2 would be especially useful [83]. This approach has been successfully employed with other environmental toxicants in larval zebrafish [84, 85]. Furthermore, exploration of Cd and Se administered at the same time may have therapeutic implications for populations where ongoing Cd exposure is unavoidable. It would be useful to quantify Cd and Se accumulation in the body and/or the cardiovascular system. Furthermore, the capability of Se to serve as a post-exposure mediation to alleviate negative cardiovascular impacts should also be further explored. Finally, the similarities in the pathophysiology of zebrafish and humans elevate the potential of zebrafish to serve as an effective model for understanding human cardiovascular disease and drug discovery.

## Supplementary Information

Below is the link to the electronic supplementary material.

Supplementary file1 (DOCX 74 KB)

## Data Availability

The data will be accessible through DryRad.

## References

[1] Timmis, A., Kazakiewicz, D., Townsend, N., Huculeci, R., Aboyans, V., & Vardas, P. (2023). Global epidemiology of acute coronary syndromes. *Nature Reviews Cardiology,**20*(11), 778–788. 10.1038/s41569-023-00884-0 37231077 10.1038/s41569-023-00884-0

[2] Kaptoge, S., Pennells, L., Bacquer, D. D., Cooney, M. T., Kavousi, M., Stevens, G., Riley, L. M., Savin, S., Khan, T., Altay, S., Amouyel, P., Assmann, G., Bell, S., Ben-Shlomo, Y., Berkman, L., Beulens, J. W., Björkelund, C., Blaha, M., Blazer, D. G., & Angelantonio, E. D. (2019). World Health Organization cardiovascular disease risk charts: Revised models to estimate risk in 21 global regions. *The Lancet Global Health,**7*(10), e1332–e1345. 10.1016/S2214-109X(19)30318-3 31488387 10.1016/S2214-109X(19)30318-3 PMC7025029

[3] Hamed, A. (2022). Air pollution and cardiovascular diseases. *Journal of Environmental Studies,**28*(1), 56–68. 10.21608/jesj.2022.181625.1036

[4] Sun, Y. (2024). Environmental correlates of mortality: How does air pollution contribute to geographic disparities in cardiovascular disease mortality? *Population and Environment,**46*(1), 1. 10.1007/s11111-023-00442-3

[5] Vos, T., Lim, S. S., Abbafati, C., Abbas, K. M., Abbasi, M., Abbasifard, M., Abbasi-Kangevari, M., Abbastabar, H., Abd-Allah, F., Abdelalim, A., Abdollahi, M., Abdollahpour, I., Abolhassani, H., Aboyans, V., Abrams, E. M., Abreu, L. G., Abrigo, M. R. M., Abu-Raddad, L. J., Abushouk, A. I., & Murray, C. J. L. (2020). Global burden of 369 diseases and injuries in 204 countries and territories, 1990–2019: A systematic analysis for the Global Burden of Disease Study 2019. *The Lancet,**396*(10258), 1204–1222. 10.1016/S0140-6736(20)30925-9 10.1016/S0140-6736(20)30925-9 PMC7567026 33069326

[6] Lamas, G. A., Bhatnagar, A., Jones, M. R., Mann, K. K., Nasir, K., Tellez-Plaza, M., Ujueta, F., & Navas-Acien, A. (2023). Contaminant metals as cardiovascular risk factors: A scientific statement from the American Heart Association. *Journal of the American Heart Association,**12*(13), e029852. 10.1161/JAHA.123.029852 37306302 10.1161/JAHA.123.029852 PMC10356104

[7] Fuller, R., Landrigan, P. J., Balakrishnan, K., Bathan, G., Bose-O’Reilly, S., Brauer, M., Caravanos, J., Chiles, T., Cohen, A., Corra, L., Cropper, M., Ferraro, G., Hanna, J., Hanrahan, D., Hu, H., Hunter, D., Janata, G., Kupka, R., Lanphear, B., & Yan, C. (2022). Pollution and health: A progress update. *The Lancet Planetary Health,**6*(6), e535–e547. 10.1016/S2542-5196(22)00090-035594895 10.1016/S2542-5196(22)00090-0

[8] Landrigan, P. J., Fuller, R., Acosta, N. J., Adeyi, O., Arnold, R., Baldé, A. B., Bertollini, R., Bose-O’Reilly, S., Boufford, J. I., & Breysse, P. N. (2018). The Lancet Commission on pollution and health. *The Lancet,**391*(10119), 462–512. 10.1016/S0140-6736(17)32345-0 29056410

[9] Satarug, S., & Phelps, K. R. (2020). *Cadmium exposure and toxicity*. CRC Press.

[10] Pinot, F., Kreps, S. E., Bachelet, M., Hainaut, P., Bakonyi, M., & Polla, B. S. (2000). Cadmium in the environment: Sources, mechanisms of biotoxicity, and biomarkers. *Reviews on Environmental Health,**15*(3), 299–324. 10.1515/REVEH.2000.15.3.299 11048333 10.1515/reveh.2000.15.3.299

[11] Agency for Toxic Substances and Disease Registry. (2022). *Substance priority list*. https://www.atsdr.cdc.gov/spl/index.html

[12] Thun, M. J., Elinder, C.-G., & Friberg, L. (1991). Scientific basis for an occupational standard for cadmium. *American Journal of Industrial Medicine,**20*(5), 629–642. 10.1002/ajim.4700200506 1793105 10.1002/ajim.4700200506

[13] Verbeeck, M., Salaets, P., & Smolders, E. (2020). Trace element concentrations in mineral phosphate fertilizers used in Europe: A balanced survey. *Science of The Total Environment,**712*, 136419. 10.1016/j.scitotenv.2019.136419 31945534 10.1016/j.scitotenv.2019.136419

[14] Satarug, S., & Moore, M. R. (2004). Adverse health effects of chronic exposure to low-level cadmium in foodstuffs and cigarette smoke. *Environmental Health Perspectives,**112*(10), 1099. 15238284 10.1289/ehp.6751 PMC1247384

[15] Jean, J., Sirot, V., Hulin, M., Le Calvez, E., Zinck, J., Noël, L., Vasseur, P., Nesslany, F., Gorecki, S., Guérin, T., & Rivière, G. (2018). Dietary exposure to cadmium and health risk assessment in children – Results of the French infant total diet study. *Food and Chemical Toxicology,**115*, 358–364. 10.1016/j.fct.2018.03.031 29580822 10.1016/j.fct.2018.03.031

[16] Wang, M., Chen, Z., Song, W., Hong, D., Huang, L., & Li, Y. (2021). A review on cadmium exposure in the population and intervention strategies against cadmium toxicity. *Bulletin of Environmental Contamination and Toxicology,**106*(1), 65–74. 10.1007/s00128-020-03088-1 33486543 10.1007/s00128-020-03088-1

[17] Satarug, S., Garrett, S. H., Sens, M. A., & Sens, D. A. (2010). Cadmium, environmental exposure, and health outcomes. *Environmental Health Perspectives,**118*(2), 182–190. 20123617 10.1289/ehp.0901234 PMC2831915

[18] Palus, J., Rydzynski, K., Dziubaltowska, E., Wyszynska, K., Natarajan, A. T., & Nilsson, R. (2003). Genotoxic effects of occupational exposure to lead and cadmium. *Mutation Research/Genetic Toxicology and Environmental Mutagenesis,**540*(1), 19–28. 10.1016/S1383-5718(03)00167-0 10.1016/s1383-5718(03)00167-0 12972055

[19] Staessen, J. A., Buchet, J.-P., Ginucchio, G., Lauwerys, R. R., Lijnen, P., Roels, H., & Fagard, R. (1996). Public health implications of environmental exposure to cadmium and lead: An overview of epidemiological studies in Belgium. *Journal of Cardiovascular Risk,**3*(1), 26–41. 10.1177/174182679600300105 8783028

[20] Kumar, S., & Sharma, A. (2019). Cadmium toxicity: Effects on human reproduction and fertility. *Reviews on Environmental Health,**34*(4), 327–338. 10.1515/reveh-2019-0016 31129655 10.1515/reveh-2019-0016

[21] Ci, J., Zhai, Y., Wang, B., Han, W., Yu, B., & An, F. (2024). Correlation between bbood cadmium levels and platelet characteristics, as well as their impact on susceptibility to coronary heart disease: Findings from NHANES 2005–2018 data. *Cardiovascular Toxicology,**24*(4), 335–344. 10.1007/s12012-024-09840-x 38448776 10.1007/s12012-024-09840-x

[22] Shin, J.-Y., Kim, J.-M., & Kim, Y. (2012). The association of heavy metals in blood, fish consumption frequency, and risk of cardiovascular diseases among Korean adults: The Korean National Health and Nutrition Examination Survey (2008–2010). *Korean Journal of Nutrition,**45*(4), 347–361. 10.4163/kjn.2012.45.4.347

[23] Tinkov, A. A., Filippini, T., Ajsuvakova, O. P., Skalnaya, M. G., Aaseth, J., Bjørklund, G., Gatiatulina, E. R., Popova, E. V., Nemereshina, O. N., Huang, P.-T., Vinceti, M., & Skalny, A. V. (2018). Cadmium and atherosclerosis: A review of toxicological mechanisms and a meta-analysis of epidemiologic studies. *Environmental Research,**162*, 240–260. 10.1016/j.envres.2018.01.008 29358116 10.1016/j.envres.2018.01.008

[24] Chen, J., Kong, A., Shelton, D., Dong, H., Li, J., Zhao, F., Bai, C., Huang, K., Mo, W., Chen, S., Xu, H., Tanguay, R. L., & Dong, Q. (2021). Early life stage transient aristolochic acid exposure induces behavioral hyperactivity but not nephrotoxicity in larval zebrafish. *Aquatic Toxicology,**238*, 105916. 10.1016/j.aquatox.2021.105916 34303159 10.1016/j.aquatox.2021.105916 PMC8881052

[25] Shelton, D. S., Dinges, Z. M., Khemka, A., Sykes, D. J., Suriyampola, P. S., Shelton, D. E. P., Boyd, P., Kelly, J. R., Bower, M., Amro, H., Glaholt, S. P., Latta, M. B., Perkins, H. L., Shaw, J. R., & Martins, E. P. (2023). A pair of cadmium-exposed zebrafish affect social behavior of the un-exposed majority. *Environmental Toxicology and Pharmacology*. 10.1016/j.etap.2023.104119 37028532 10.1016/j.etap.2023.104119 PMC10423439

[26] Shelton, D. S., Suriyampola, P. S., Dinges, Z. M., Glaholt, S. P., Shaw, J. R., & Martins, E. P. (2024). Plants buffer some of the effects of a pair of cadmium-exposed zebrafish on the un-exposed majority. *Environmental Toxicology and Pharmacology,**107*, 104419. 10.1016/j.etap.2024.104419 38508506 10.1016/j.etap.2024.104419 PMC11042042

[27] Staudt, D., & Stainier, D. (2012). Uncovering the molecular and cellular mechanisms of heart development using the zebrafish. *Annual Review of Genetics,**46*, 397–418. 10.1146/annurev-genet-110711-155646 22974299 10.1146/annurev-genet-110711-155646 PMC6982417

[28] Kelly, J. R., Shelton, S. G., Daniel, D. K., Bhat, A., Mondal, R., Nipple, F., Amro, H., Bower, M. E., Isaac, G., McHaney, G., Martins, E. P., & Shelton, D. S. (2021). Wild zebrafish sentinels: Biological monitoring of site differences using behavior and morphology. *Toxics*. 10.3390/toxics9070165 34357908 10.3390/toxics9070165 PMC8309768

[29] Schlenker, L. S., Faillettaz, R., Stieglitz, J. D., Lam, C. H., Hoenig, R. H., Cox, G. K., Heuer, R. M., Pasparakis, C., Benetti, D. D., Paris, C. B., & Grosell, M. (2021). Remote predictions of mahi-mahi (*Coryphaena hippurus*) spawning in the open ocean using summarized accelerometry data. *Frontiers in Marine Science*. 10.3389/fmars.2021.626082

[30] Shelton, D. S., Shelton, S. G., Daniel, D. K., Raja, M., Bhat, A., Tanguay, R. L., Higgs, D. M., & Martins, E. P. (2020). Collective behavior in wild zebrafish. *Zebrafish,**17*(4), 243–252. 32513074 10.1089/zeb.2019.1851 PMC7869874

[31] Suriyampola, P. S., Shelton, D. S., Shukla, R., Roy, T., Bhat, A., & Martins, E. P. (2016). Zebrafish social behavior in the wild. *Zebrafish,**13*(1), 1–8. 10.1089/zeb.2015.1159 26671510 10.1089/zeb.2015.1159

[32] Heideman, W., Antkiewicz, D. S., Carney, S. A., & Peterson, R. E. (2005). Zebrafish and cardiac toxicology. *Cardiovascular Toxicology,**5*(2), 203–214. 10.1385/CT:5:2:203 16046794 10.1385/ct:5:2:203

[33] Perrichon, P., Grosell, M., & Burggren, W. W. (2017). Heart performance determination by visualization in larval fishes: Influence of alternative models for heart shape and volume. *Frontiers in Physiology*. 10.3389/fphys.2017.00464 28725199 10.3389/fphys.2017.00464 PMC5495860

[34] Hofsteen, P., Plavicki, J., Johnson, S. D., Peterson, R. E., & Heideman, W. (2013). Sox9b is required for epicardium formation and plays a role in TCDD-induced heart malformation in zebrafish. *Molecular Pharmacology,**84*(3), 353–360. 10.1124/mol.113.086413 23775563 10.1124/mol.113.086413 PMC3876814

[35] Avallone, B., Crispino, R., Cerciello, R., Simoniello, P., Panzuto, R., & Maria Motta, C. (2015). Cadmium effects on the retina of adult *Danio rerio*. *Comptes Rendus Biologies,**338*(1), 40–47. 10.1016/j.crvi.2014.10.005 25528674 10.1016/j.crvi.2014.10.005

[36] Wold, M., Beckmann, M., Poitra, S., Espinoza, A., Longie, R., Mersereau, E., Darland, D. C., & Darland, T. (2017). The longitudinal effects of early developmental cadmium exposure on conditioned place preference and cardiovascular physiology in zebrafish. *Aquatic Toxicology,**191*, 73–84. 10.1016/j.aquatox.2017.07.017 28804037 10.1016/j.aquatox.2017.07.017 PMC5764186

[37] Alsop, D., & Wood, C. M. (2011). Metal uptake and acute toxicity in zebrafish: Common mechanisms across multiple metals. *Aquatic Toxicology,**105*(3), 385–393. 10.1016/j.aquatox.2011.07.010 21820385 10.1016/j.aquatox.2011.07.010

[38] Cheng, S. H., Wai, A. W. K., So, C. H., & Wu, R. S. S. (2000). Cellular and molecular basis of cadmium-induced deformities in zebrafish embryos. *Environmental Toxicology and Chemistry,**19*(12), 3024–3031. 10.1002/etc.5620191223

[39] Gao, Y., Feng, J., & Zhu, L. (2015). Prediction of acute toxicity of cadmium and lead to zebrafish larvae by using a refined toxicokinetic-toxicodynamic model. *Aquatic Toxicology,**169*, 37–45. 10.1016/j.aquatox.2015.09.005 26513221 10.1016/j.aquatox.2015.09.005

[40] Mitovic, N., Maksimovic, S., Puflovic, D., Kovacevic, S., Lopicic, S., Todorovic, J., Spasic, S., Dincic, M., & Ostojic, J. N. (2021). Cadmium significantly changes major morphometrical points and cardiovascular functional parameters during early development of zebrafish. *Environmental Toxicology and Pharmacology,**87*, 103723. 10.1016/j.etap.2021.103723 34391906 10.1016/j.etap.2021.103723

[41] Yin, J., Yang, J., Zhang, F., Miao, P., Lin, Y., & Chen, M. (2014). Individual and joint toxic effects of cadmium sulfate and α-naphthoflavone on the development of zebrafish embryo. *Journal of Zhejiang University. Science. B,**15*(9), 766–775. 10.1631/jzus.B1400091 25183031 10.1631/jzus.B1400091 PMC4162878

[42] Liu, P., Zhao, Y., Wang, S., Xing, H., & Dong, W.-F. (2021). Effect of combined exposure to silica nanoparticles and cadmium chloride on female zebrafish ovaries. *Environmental Toxicology and Pharmacology,**87*, 103720. 10.1016/j.etap.2021.103720 34332080 10.1016/j.etap.2021.103720

[43] Chen, J., Pan, T., Wan, N., Sun, Z., Zhang, Z., & Li, S. (2017). Cadmium-induced endoplasmic reticulum stress in chicken neutrophils is alleviated by selenium. *Journal of Inorganic Biochemistry,**170*, 169–177. 10.1016/j.jinorgbio.2017.02.022 28249225 10.1016/j.jinorgbio.2017.02.022

[44] Li, J.-L., Jiang, C.-Y., Li, S., & Xu, S.-W. (2013). Cadmium induced hepatotoxicity in chickens (*Gallus domesticus*) and ameliorative effect by selenium. *Ecotoxicology and Environmental Safety,**96*, 103–109. 10.1016/j.ecoenv.2013.07.007 23906702 10.1016/j.ecoenv.2013.07.007

[45] Liu, R., Jia, T., Cui, Y., Lin, H., & Li, S. (2018). The protective effect of selenium on the chicken pancreas against cadmium toxicity via alleviating oxidative stress and autophagy. *Biological Trace Element Research,**184*(1), 240–246. 10.1007/s12011-017-1186-9 28994040 10.1007/s12011-017-1186-9

[46] Liu, S., Xu, F., Yang, Z., Li, M., Min, Y., & Li, S. (2014). Cadmium-induced injury and the ameliorative effects of selenium on chicken splenic lymphocytes: Mechanisms of oxidative stress and apoptosis. *Biological Trace Element Research,**160*(3), 340–351. 10.1007/s12011-014-0070-0 25035189 10.1007/s12011-014-0070-0

[47] Tan, S., Chi, Q., Liu, T., Sun, Z., Min, Y., Zhang, Z., & Li, S. (2017). Alleviation mechanisms of selenium on cadmium-spiked neutrophil injury to chicken. *Biological Trace Element Research,**178*(2), 301–309. 10.1007/s12011-016-0924-8 28064415 10.1007/s12011-016-0924-8

[48] Burk, R. F., & Hill, K. E. (2015). Regulation of selenium metabolism and transport. *Annual Review of Nutrition,**35*, 109–134. 10.1146/annurev-nutr-071714-034250 25974694 10.1146/annurev-nutr-071714-034250

[49] Castellano, S., Lobanov, A. V., Chapple, C., Novoselov, S. V., Albrecht, M., Hua, D., Lescure, A., Lengauer, T., Krol, A., Gladyshev, V. N., & Guigó, R. (2005). Diversity and functional plasticity of eukaryotic selenoproteins: Identification and characterization of the SelJ family. *Proceedings of the National Academy of Sciences,**102*(45), 16188–16193. 10.1073/pnas.0505146102 10.1073/pnas.0505146102 PMC1283428 16260744

[50] Whanger, P. D. (2002). Selenocompounds in plants and animals and their biological significance. *Journal of the American College of Nutrition,**21*(3), 223–232. 10.1080/07315724.2002.10719214 12074249 10.1080/07315724.2002.10719214

[51] Lu, J., & Holmgren, A. (2009). Selenoproteins *. *Journal of Biological Chemistry,**284*(2), 723–727. 10.1074/jbc.R800045200 18757362 10.1074/jbc.R800045200

[52] Reeves, M. A., & Hoffmann, P. R. (2009). The human selenoproteome: Recent insights into functions and regulation. *Cellular and Molecular Life Sciences,**66*(15), 2457–2478. 10.1007/s00018-009-0032-4 19399585 10.1007/s00018-009-0032-4 PMC2866081

[53] Cuypers, A., Plusquin, M., Remans, T., Jozefczak, M., Keunen, E., Gielen, H., Opdenakker, K., Nair, A. R., Munters, E., Artois, T. J., Nawrot, T., Vangronsveld, J., & Smeets, K. (2010). Cadmium stress: An oxidative challenge. *BioMetals,**23*(5), 927–940. 10.1007/s10534-010-9329-x 20361350 10.1007/s10534-010-9329-x

[54] Forman, H. J., & Zhang, H. (2021). Targeting oxidative stress in disease: Promise and limitations of antioxidant therapy. *Nature Reviews Drug Discovery,**20*(9), 689–709. 10.1038/s41573-021-00233-1 34194012 10.1038/s41573-021-00233-1 PMC8243062

[55] Liu, J., Qu, W., & Kadiiska, M. B. (2009). Role of oxidative stress in cadmium toxicity and carcinogenesis. *Toxicology and Applied Pharmacology,**238*(3), 209–214. 10.1016/j.taap.2009.01.029 19236887 10.1016/j.taap.2009.01.029 PMC4287357

[56] Valko, M., Rhodes, C. J., Moncol, J., Izakovic, M., & Mazur, M. (2006). Free radicals, metals and antioxidants in oxidative stress-induced cancer. *Chemico-Biological Interactions,**160*(1), 1–40. 10.1016/j.cbi.2005.12.009 16430879 10.1016/j.cbi.2005.12.009

[57] Zwolak, I., & Zaporowska, H. (2012). Selenium interactions and toxicity: A review. *Cell Biology and Toxicology,**28*(1), 31–46. 10.1007/s10565-011-9203-9 21913064 10.1007/s10565-011-9203-9

[58] Zwolak, I. (2020). The role of selenium in arsenic and cadmium toxicity: An updated review of scientific literature. *Biological Trace Element Research,**193*(1), 44–63. 10.1007/s12011-019-01691-w 30877523 10.1007/s12011-019-01691-w PMC6914719

[59] Dolgova, N. V., Hackett, M. J., MacDonald, T. C., Nehzati, S., James, A. K., Krone, P. H., George, G. N., & Pickering, I. J. (2016). Distribution of selenium in zebrafish larvae after exposure to organic and inorganic selenium forms. *Metallomics,**8*(3), 305–312. 10.1039/c5mt00279f 26781816 10.1039/c5mt00279f

[60] Cai, J., Zhang, Y., Yang, J., Liu, Q., Zhao, R., Hamid, S., Wang, H., Xu, S., & Zhang, Z. (2017). Antagonistic effects of selenium against necroptosis injury via adiponectin-necrotic pathway induced by cadmium in heart of chicken. *RSC Advances,**7*(70), 44438–44446. 10.1039/C7RA07952D

[61] Feng, J., Yang, F., Wu, H., Xing, C., Xue, H., Zhang, L., Zhang, C., Hu, G., & Cao, H. (2022). Selenium protects against cadmium-induced cardiac injury by attenuating programmed cell death via PI3K/AKT/PTEN signaling. *Environmental Toxicology,**37*(5), 1185–1197. 10.1002/tox.23475 35099092 10.1002/tox.23475

[62] Shalihat, A., Hasanah, A. N., Lesmana, R., Budiman, A., & Gozali, D. (2021). The role of selenium in cell survival and its correlation with protective effects against cardiovascular disease: A literature review. *Biomedicine & Pharmacotherapy,**134*, 111125. 33341057 10.1016/j.biopha.2020.111125

[63] Alexanian, I., Parissis, J., Farmakis, D., Pantziou, C., Ikonomidis, I., Paraskevaidis, I., Ioannidou, S., Sideris, A., Kremastinos, D., Lekakis, J., & Filippatos, G. (2014). Selenium contributes to myocardial injury and cardiac remodeling in heart failure. *International Journal of Cardiology,**176*(1), 272–273. 10.1016/j.ijcard.2014.06.095 25042657 10.1016/j.ijcard.2014.06.095

[64] Al-Mubarak, A. A., van der Meer, P., & Bomer, N. (2021). Selenium, selenoproteins, and heart failure: Current knowledge and future perspective. *Current Heart Failure Reports,**18*(3), 122–131. 10.1007/s11897-021-00511-4 33835398 10.1007/s11897-021-00511-4 PMC8163712

[65] Flores-Mateo, G., Navas-Acien, A., Pastor-Barriuso, R., & Guallar, E. (2006). Selenium and coronary heart disease: A meta-analysis2. *The American Journal of Clinical Nutrition,**84*(4), 762–773. 10.1093/ajcn/84.4.762 17023702 10.1093/ajcn/84.4.762 PMC1829306

[66] Rayman, M. P., Stranges, S., Griffin, B. A., Pastor-Barriuso, R., & Guallar, E. (2011). Effect of supplementation with high-selenium yeast on plasma lipids. *Annals of Internal Medicine,**154*(10), 656–665. 10.7326/0003-4819-154-10-201105170-00005 21576533 10.7326/0003-4819-154-10-201105170-00005

[67] Kim, K., Melough, M. M., Vance, T. M., Noh, H., Koo, S. I., & Chun, O. K. (2019). Dietary cadmium intake and sources in the US. *Nutrients*, *11*(1), Article 1. 10.3390/nu11010002 10.3390/nu11010002 PMC6356330 30577418

[68] Shaw, R. G., & Mitchell-Olds, T. (1993). Anova for unbalanced data: An overview. *Ecology,**74*(6), 1638–1645. 10.2307/1939922

[69] R Core Team. (2015). *R: A language and environment for statistical computing. R Foundation for Statistical Computing, Vienna, Austria.* http://www.R-project.org/

[70] Diaz, D., Ujueta, F., Mansur, G., Lamas, G. A., Navas-Acien, A., & Arenas, I. A. (2021). Low-level cadmium exposure and atherosclerosis. *Current Environmental Health Reports,**8*(1), 42–53. 10.1007/s40572-021-00304-w 33754286 10.1007/s40572-021-00304-w

[71] Ge, J., Guo, K., Zhang, C., Talukder, M., Lv, M.-W., Li, J.-Y., & Li, J.-L. (2021). Comparison of nanoparticle-selenium, selenium-enriched yeast and sodium selenite on the alleviation of cadmium-induced inflammation via NF-kB/IκB pathway in heart. *Science of The Total Environment,**773*, 145442. 10.1016/j.scitotenv.2021.145442 33940727 10.1016/j.scitotenv.2021.145442

[72] Jamall, I. S., Naik, M., Sprowls, J. J., & Trombetta, L. D. (1989). A comparison of the effects of dietary cadmium on heart and kidney antioxidant enzymes: Evidence for the greater vulnerability of the heart to cadmium toxicity. *Journal of Applied Toxicology,**9*(5), 339–345. 10.1002/jat.2550090510 2592733 10.1002/jat.2550090510

[73] Lin, Y., Hu, L., Li, X., Ma, J., Li, Q., Yuan, X., & Zhang, Y. (2024). The beneficial and toxic effects of selenium on zebrafish. A systematic review of the literature. *Toxicology Research,**13*(2), 062. 10.1093/toxres/tfae062 PMC11031411 38645626

[74] Lippman, S. M., Klein, E. A., Goodman, P. J., Lucia, M. S., Thompson, I. M., Ford, L. G., Parnes, H. L., Minasian, L. M., Gaziano, J. M., Hartline, J. A., Parsons, J. K., Bearden, J. D., Crawford, E. D., Goodman, G. E., Claudio, J., Winquist, E., Cook, E. D., Karp, D. D., Walther, P., & Coltman, C. A. (2009). Effect of selenium and vitamin E on risk of prostate cancer and other cancers: The selenium and vitamin E cancer prevention trial (SELECT). *JAMA,**301*(1), 39–51. 10.1001/jama.2008.864 19066370 10.1001/jama.2008.864 PMC3682779

[75] Stranges, S., Marshall, J. R., Trevisan, M., Natarajan, R., Donahue, R. P., Combs, G. F., Farinaro, E., Clark, L. C., & Reid, M. E. (2006). Effects of selenium supplementation on cardiovascular disease incidence and mortality: Secondary analyses in a randomized clinical trial. *American Journal of Epidemiology,**163*(8), 694–699. 10.1093/aje/kwj097 16495471 10.1093/aje/kwj097

[76] Vinceti, M., Filippini, T., & Wise, L. A. (2018). Environmental selenium and human health: An update. *Current Environmental Health Reports,**5*(4), 464–485. 10.1007/s40572-018-0213-0 30280317 10.1007/s40572-018-0213-0

[77] Stoffaneller, R., & Morse, N. L. (2015). A review of dietary selenium intake and selenium status in Europe and the Middle East. *Nutrients*, *7*(3), Article 3. 10.3390/nu7031494 10.3390/nu7031494 PMC4377864 25734564

[78] Thomson, C. D. (2004). Assessment of requirements for selenium and adequacy of selenium status: A review. *European Journal of Clinical Nutrition,**58*(3), 391–402. 10.1038/sj.ejcn.1601800 14985676 10.1038/sj.ejcn.1601800

[79] Wasowicz, W., Gromadzinska, J., Rydzynski, K., & Tomczak, J. (2003). Selenium status of low-selenium area residents: Polish experience. *Toxicology Letters,**137*(1), 95–101. 10.1016/S0378-4274(02)00383-1 12505435 10.1016/s0378-4274(02)00383-1

[80] Dong, X., Deng, Y., & Chen, G. (2024). Selenium intake in relation to all-cause and cardiovascular mortality in individuals with nonalcoholic fatty liver disease: A nationwide study in nutrition. *PLOS ONE,**19*(5), e0303140. 10.1371/journal.pone.0303140 38768120 10.1371/journal.pone.0303140 PMC11104653

[81] Heuer, R. M., Galli, G. L. J., Shiels, H. A., Fieber, L. A., Cox, G. K., Mager, E. M., Stieglitz, J. D., Benetti, D. D., Grosell, M., & Crossley Ii, D. A. (2019). Impacts of *Deepwater Horizon* crude OIL on Mahi-Mahi (*Coryphaena hippurus*) heart cell function. *Environmental Science & Technology,**53*(16), 9895–9904. 10.1021/acs.est.9b03798 31343865 10.1021/acs.est.9b03798

[82] Nguyen, N. H. A., & Falagan-Lotsch, P. (2023). Mechanistic insights into the biological effects of engineered nanomaterials: A focus on gold nanoparticles. *International Journal of Molecular Sciences*, *24*(4), Article 4. 10.3390/ijms24044109 10.3390/ijms24044109 PMC9963226 36835521

[83] Gupta, V., Gemberling, M., Karra, R., Rosenfeld, G. E., Evans, T., & Poss, K. D. (2013). An injury-responsive gata4 program shapes the zebrafish cardiac ventricle. *Current Biology,**23*(13), 1221–1227. 23791730 10.1016/j.cub.2013.05.028 PMC3759223

[84] Diao, W., Yan, J., Wang, X., Qian, Q., & Wang, H. (2023). Mechanisms regarding cardiac toxicity triggered by up-regulation of miR-144 in larval zebrafish upon exposure to triclosan. *Journal of Hazardous Materials,**443*, 130297. 36368065 10.1016/j.jhazmat.2022.130297

[85] Wan, M., Liu, J., Yang, D., Xiao, Z., Li, X., Liu, J., Huang, L., Liu, F., Zhang, S., & Tao, Q. (2024). Dimethyl fumarate induces cardiac developmental toxicity in zebrafish via down-regulation of oxidative stress. *Toxicology,**503*, 153735. 38272385 10.1016/j.tox.2024.153735

