# Supplementary material for: Therapeutic Efficacy of Selenium Pre-treatment in Mitigating Cadmium-Induced Cardiotoxicity in Zebrafish (Danio rerio)
Source: Cardiovasc Toxicol. 2024 Aug 30;24(11):1287–300. doi: 10.1007/s12012-024-09910-0 (PMC11445284; doi:10.1007/s12012-024-09910-0)
Supplement: Supplementary file 1 — Supplementary file1 (DOCX 74 KB) [file 12012_2024_9910_MOESM1_ESM.docx]

Supplementary Information


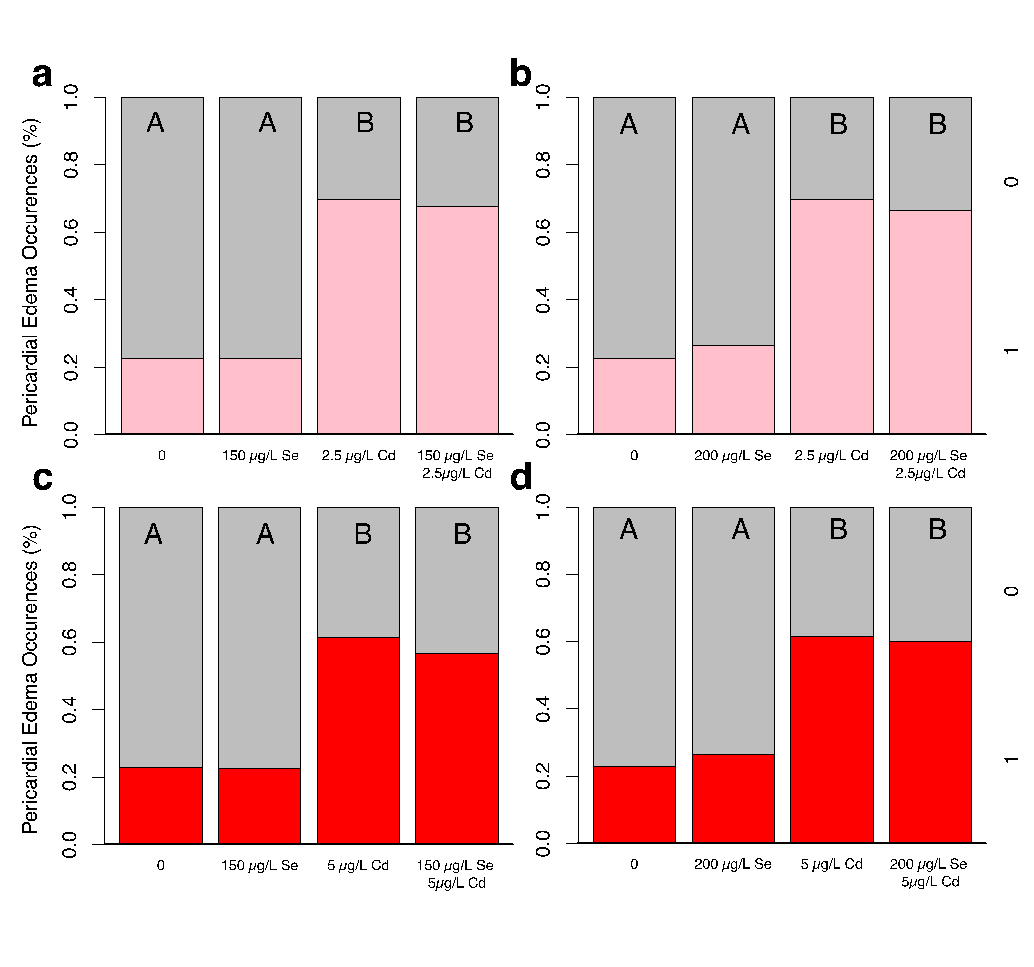


Fig S1. Fish exposed to various concentrations of Se and Cd. a) The presence of pericardial edemas in fish exposed to E3 media (or control; *n* = 167), 2.5 µg/L of Cd (*n* = 169), 150 µg/L of Se (*n* = 128) and a pre-treatment of 150 µg/L of Se prior to a 2.5 µg/L Cd exposure (*n* = 130). Fish exposed to 150 µg/L of Se prior to Cd and those 2.5 µg/L of Cd show nearly three times as many incidences of pericardial edemas as those exposed to 150 µg/L of Se and E3 media (control). Fish exposed to 150 µg/L of Se prior to Cd and those exposed to 2.5 µg/L of Cd show similar incidences of pericardial edemas. Control fish and those exposed to 150 µg/L of Se show similar incidences of pericardial edemas. This led to a significant difference across treatment groups, *H* (3, *n* = 594) = 127.45, *p* < 0.0001. b) The presence of pericardial edemas in fish exposed to E3 media (or control; *n* = 167), 2.5 µg/L of Cd (*n* = 169), 200 µg/L of Se (*n* = 128) and a pre-treatment of 200 µg/L of Se prior to a 2.5 µg/L Cd exposure (*n* = 128). Fish exposed to 200 µg/L of Se prior to Cd and those 2.5 µg/L of Cd show at least 2.5 times as many incidences of pericardial edemas as those exposed to 200 µg/L of Se and control fish. Fish exposed to 200 µg/L of Se prior to 2.5 µg/L of Cd and those exposed to 2.5 µg/L of Cd show similar incidences of pericardial edemas. Se deficient fish and those exposed to 200 µg/L of Cd show similar incidences of pericardial edemas, *H*(3, *n*= 592) = 115.46, *p* < 0.0001. c) The presence of pericardial edemas in fish exposed to E3 media (or control; *n* = 167), 5 µg/L of Cd (*n* = 166), 150 µg/L of Se (*n* = 128) and a pre-treatment of 150 µg/L of Se prior to a 5 µg/L Cd exposure (*n* = 132). Fish exposed to 150 µg/L of Se prior to Cd and those 5 µg/L of Cd show at least 2.5 times as many incidences of pericardial edemas as those exposed to 150 µg/L of Se and E3 media (control). Fish exposed to 150 µg/L of Se prior to 5 µg/L of Cd and those exposed to 5 µg/L of Cd show similar incidences of pericardial edemas. Control fish (*n* = 167) and those exposed to 150 µg/L of Se (n = 128) show similar incidences of pericardial edemas (*H*(3, n = 593) = 82.89, *p* < 0.0001). d) The presence of pericardial edemas in fish exposed to E3 media (or control; *n* = 167), 5 µg/L of Cd (*n* = 166), 200 µg/L of Se (*n* = 128) and a pre-treatment of 200 µg/L of Se prior to a 5 µg/L Cd exposure (*n* = 128). Fish exposed to 200 µg/L of Se prior to Cd and those 2.5 µg/L of Cd show at least 2.2 times as many incidences of pericardial edemas as those exposed to 200 µg/L of Se and control fish. Fish exposed to 200 µg/L of Se prior to Cd and those exposed to 5 µg/L of Cd show similar incidences of pericardial edemas. Control fish and those exposed to 200 µg/L of Se show similar incidences of pericardial edemas. This led to a significant difference across treatments, *H*(3, *n* = 589) = 80.46, *p* < 0.0001.
